# Recent advances and challenges in the use of CRISPR/Cas9 genome editing for understanding neuronal cell biology

**DOI:** 10.1117/1.NPh.10.4.044403

**Published:** 2023-06-15

**Authors:** Harold D. MacGillavry

**Affiliations:** Utrecht University, Division of Cell Biology, Neurobiology and Biophysics, Department of Biology, Faculty of Science, The Netherlands

**Keywords:** genome editing, CRISPR/Cas9, fluorescence microscopy, neurons, knock-in

## Abstract

The ability to accurately map and manipulate the dynamic subcellular distribution of proteins is key for a mechanistic understanding of neuronal functioning. Current fluorescence microscopy techniques provide access to subcellular protein organization at increasing resolution but are often restricted by the availability of methods that reliably label endogenous proteins. Excitingly, recent development in CRISPR/Cas9 genome editing now allows researchers to specifically tag and visualize endogenous proteins, overcoming limitations associated with current labeling strategies. This article will discuss the progress that has been made in the last years that has led to the development of CRISPR/Cas9 genome editing tools for the reliable mapping of endogenous proteins in neurons. Furthermore, recently developed tools enable the duplex labeling of two proteins simultaneously and acute manipulation of protein distribution. Future implementations of this generation of genome editing technologies will undoubtedly drive progress in molecular and cellular neurobiology.

## 1 Background

Neurons are highly compartmentalized cells, and the correct subcellular distribution of neuronal proteins is key for the proper execution of neuronal functions. Mapping the dynamic subcellular organization of proteins at high precision is therefore essential for our understanding of neuronal functioning.^1^ Excitingly, the rapid developments in the field of super-resolution microscopy continue to increase the resolving power of microscopes, providing unique new insights in neuronal cell biology. However, the ever-increasing improvements in microscopy put a strong demand on methods used to label molecular complexes. Currently common labeling approaches, such as overexpression of tagged proteins and antibody labeling have several shortcomings that can be overcome by tagging endogenous proteins in cells with CRISPR/Cas9-based genome editing. Due to its simplicity, versatility, and precision, CRISPR/Cas9-based genome editing techniques have rapidly revolutionized biological research.^2^ This article will discuss how CRISPR/Cas9-based methodologies can be exploited to accurately label and manipulate endogenous proteins in neurons, overcoming many of the limitations associated with current labeling strategies.

## 2 CRISPR/Cas9 Technology for Endogenous Protein Tagging in Neurons

**Fig. 1:**
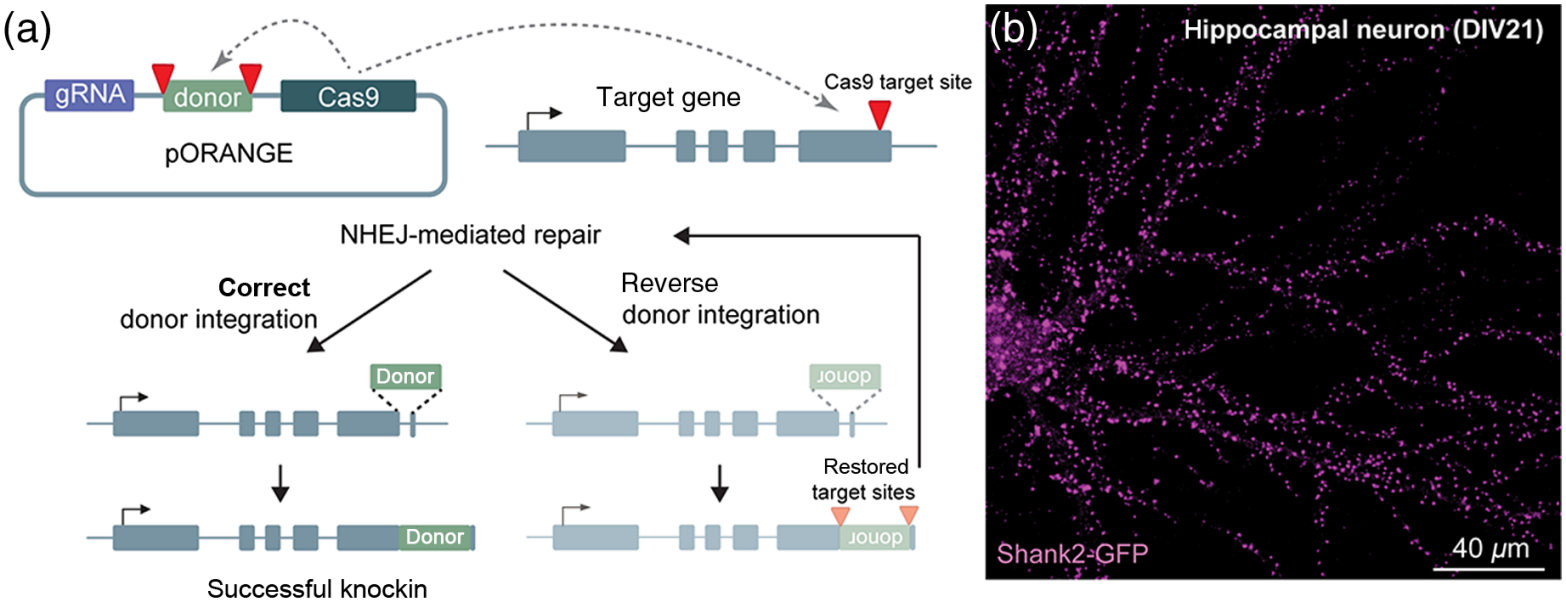
Mechanism of ORANGE-mediated epitope tagging of endogenous proteins in neurons. (a) The pORANGE knock-in construct expresses Cas9 and a 20-bp gRNA sequence and contains the donor sequence encoding an epitope tag flanked by target sequences identical to the genomic target sequence. The gRNA will guide Cas9 to target both the genomic locus of interest and the donor sequence to release the repair sequence from the plasmid. After cleavage, the donor sequence released from the plasmid is integrated in the genome by NHEJ-mediated repair. Importantly, the orientation of the target sequence and PAM sites flanking the donor is inverted compared with the genomic sequence so that inverted integration of the donor sequence will lead to removal by Cas9 and a new round of repair can take place. (b) Example of a Shank2-GFP knock-in neuron stained with anti-GFP to amplify the signal.

CRISPR/Cas9-based genome editing techniques allow the site-specific modification, removal, or addition of DNA segments. CRISPR/Cas9 relies on a guide RNA (gRNA) sequence containing a protospacer adjacent motif (PAM) that instructs Cas9 to induce a double-stranded DNA break (DSB) at a specified site in the genome. This DSB can trigger two endogenous DNA repair pathways: the non-homologous end-joining (NHEJ) pathway and the homology-directed repair (HDR) pathway. The HDR pathway can be utilized to direct a precise recombination event between a homologous donor sequence and the sequence around the DSB to generate site-specific knock-ins. HDR is however tightly controlled by the cell cycle and is strongly suppressed in post-mitotic cells, such as neurons. Nevertheless, HDR has been used successfully in neurons to generate knock-ins and visualize endogenously tagged proteins.^3^^–^^6^ In contrast, NHEJ-mediated genome editing is much more efficient than HDR in non-dividing cells.^7^ Even though NHEJ was long considered to be an inherently error-prone process, the homology-independent targeted integration (HITI) method demonstrated that NHEJ-based integration of donor sequences can be highly efficient (∼4-fold more efficient compared to HDR) with minimal mutagenesis and off-target integration.^7^ Based on HITI, we developed a versatile genome editing toolbox that we termed ORANGE: open resource for neuronal genome editing^8^ (Fig. 1). We designed a simple vector system and generated a library of knock-in vectors labeling a broad range of synaptic, trafficking, and signaling proteins in neurons. Importantly, we found that tagging endogenous proteins omitted limitations associated with overexpression or immunolabeling. For instance, PSD-95 overexpression is known to severely affect synapse size and function.^9^^–^^12^ We found that overexpressed PSD-95 was not only targeted to synapses but also accumulated in dendritic shafts and led to a significant increase in synapse size. Endogenously labeled PSD-95 using ORANGE faithfully labeled synapses without such side-effects. Moreover, we were able to tag specific receptor isoforms, resolve live-cell dynamics of endogenous proteins, such as actin and CaMKII, and track surface diffusion of endogenous glutamate receptors. Using viral vectors, we and others further demonstrated the broad application of this technique in different neuronal preparations, including organotypic slices and *in vivo* mouse brain.^8^^,^^13^ In recent adaptations, other groups used two gRNAs to replace entire exons^14^^,^^15^ or introduced synthetic exons in intronic regions to generate fusion proteins.^16^^–^^18^ Since these methods target intronic regions, these methods prevent indel generation in coding sequences. Labeling efficiencies vary widely with both NHEJ- and HDR-based approaches for different targets and using different delivery methods. Generally, HDR-based approaches label a small population (<5%) of the targeted neurons.^3^^,^^6^ NHJE-mediated approaches typically label 5 - 20% of the targeted neurons,^7^^,^^13^ and intron-targeting methods can reach up to ∼45%.^14^^,^^16^ Continuing developments will increase the versatility, efficiency and specificity of genome-editing techniques for protein tagging in neurons.

## 3 Multiplexing and Manipulation of Endogenous Proteins

**Fig. 2:**
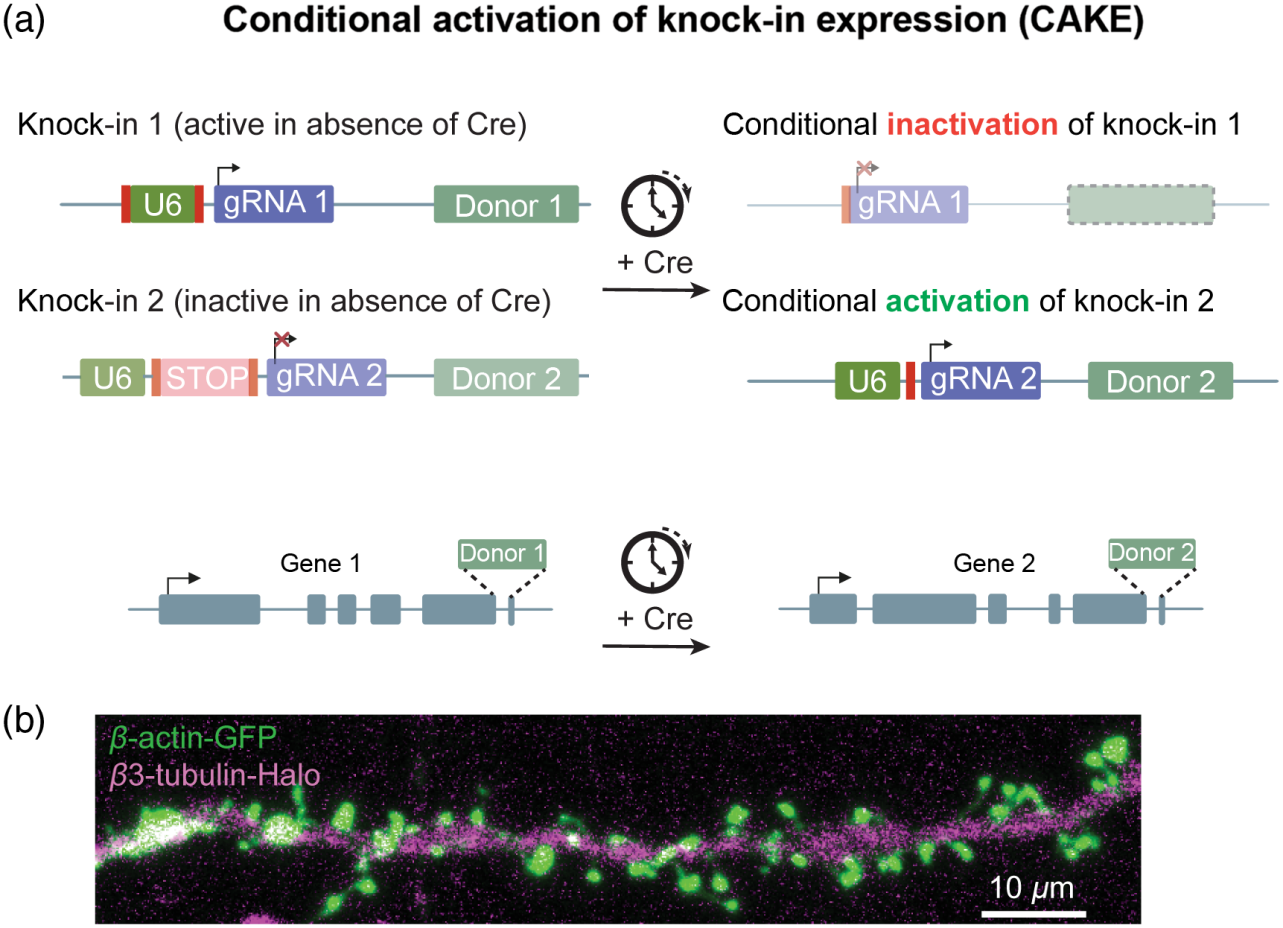
Mechanism of duplex labeling using CAKE. (a) CAKE uses two knock-in plasmids that are controlled by Cre recombinase. In the absence of Cre, only the knock-in cassette targeting gene 1 is active. After a delay in time to complete the first round of genome editing, Cre expression is experimentally induced (by viral delivery or tamoxifen-induced expression). Cre then simultaneously inactivates the expression of the gRNA from knock-in plasmid 1 and activates gRNA expression from knock-in plasmid 2. Using this strategy, two knock-in events at two different genes can be separated over time. (b) Example image shows a double-knock-in neuron expressing GFP-tagged actin and Halo-tagged tubulin.

Multiplexing genome editing to tag two or more proteins simultaneously could allow for co-localization or interaction studies using for instance Förster resonance energy transfer. Multiplexing remained challenging however, because in NHEJ-based repair the donor sequence can be integrated in any DSB. Thus, when editing two loci simultaneously, the integration of specific donor sequences is untargeted, leading to crosstalk. One solution that was shown to generate double knock-ins^13^ is to create donor sequences that prevent protein labeling when inserted in the incorrect gene. This design however restricts the choice for the location of the protein tag, and indels or integration of the incorrect donor may generate null mutations. We therefore developed an extension of the ORANGE toolbox: conditional activation of knock-in expression (CAKE) that essentially separates two genome editing events over time to direct the sequential integration of tags (Fig. 2). In an initial implementation, we designed a dual-vector system where the first knock-in construct integrates a C-terminal GFP-P2A-Cre recombinase such that a GFP fusion protein and recombinase are expressed simultaneously. The second construct contained a Cre-dependent Lox-STOP-Lox sequence in the U6 promoter, preventing expression of the gRNA until Cre is expressed. Thus, only when the first integration event was successful and the fusion protein was expressed, the second knock-in construct would be activated to drive integration of another donor in the second target gene. This idea was successful^8^ but we noticed that expression levels of the first fusion protein were sometimes reduced, and we occasionally found cross-talk events. To overcome these limitations, we redesigned the approach and placed both knock-in constructs under the bidirectional control of a Cre-lox switch, such that Cre recombinase expression turns off the first knock-in and turns on the second knock-in. In addition, we found that the timed expression of Cre by viral infection or tamoxifen-controlled Cre expression worked very efficiently.^19^ This improved the control over the separation of the genome editing events over time and ensures proper integration of the tags in the correct target gene. The efficiency of duplex labelling varied significantly between different target sequences but also seemed to depend on the amount of donor DNA and timing of Cre expression.

Reliable labeling of endogenous proteins in neurons is an important step, but for a mechanistic understanding of neuronal cell biology, tools are required that can precisely manipulate the abundance or localization of proteins. Targeted CRISPR/Cas9-mediated knock-out is relatively simple and effective and can be used to study protein function in neurons.^20^^,^^21^ Genetic fusion of conditional degradation motifs or degrons could be a powerful approach to induce rapid degradation of targeted proteins.^22^ To acutely reposition endogenous proteins, we exploited the multiplexing system to implement an inducible rapalog-based dimerization system. In this chemical dimerization system, the interaction between two proteins, one tagged with FKBP and the other with FRB, can be induced by addition of rapalog, a synthetic analogue of rapamycin. Using this system, we could acutely immobilize endogenous GluA1-containing AMPA receptors at the postsynaptic density.^19^ This system thus allows for acute control over endogenous protein distribution and dynamics, providing new ways to interrogate the molecular mechanisms that control neuronal functioning.

## 4 Concluding Remarks

The exciting developments in the field of genome editing continue to drive progress in neuronal cell biology. Nevertheless, several improvements could be made to increase the utility of genome editing. First, the efficiency of genome editing in neurons is still relatively low. We found that the efficiency of individual knock-in vectors targeting the same gene, even targeting PAM sites spaced by only a few base pairs, could differ ∼5-fold.^8^ However, relating these efficiencies to prediction scores yielded little insights. Thus, better understanding of what factors determine the integration efficiency, e.g., gRNA sequence, properties of Cas9, and local chromatin status of the target locus, would be valuable for further optimization of CRISPR/Cas9 tagging. Second, as with any other labeling technique, epitope tagging can adversely affect the localization and function of endogenous proteins. This should always be thoroughly assessed through comparative localization studies and functional characterization. Improvement in fluorescent tagging technologies will hopefully provide smaller and brighter tags to reliably detect endogenous proteins. All in all, while further optimization and validation of CRISPR technologies are needed, these tools provide a plethora of new possibilities for neuronal cell biologists. Particularly, the ability to not only tag but also manipulate the levels and positioning of endogenous components is a powerful tool to delineate a mechanistic understanding of subcellular processes in neurons.
